# Health Rules

**Published:** 1877-07

**Authors:** 


If you are well, let yourself alone.

If you are not well, make no change in any of your habits without consulting an educated physician.

Do not take it for granted that any habit or practice is advisable for *you*, simply because *others* have health in their observance. But if you want to make the experiment, *first place yourself in all their conditions.*

